# Supplementary material for: Accessing mental health care: A population-level exploration of the impact of immigration duration in the United States 2019–2023
Source: PLOS Ment Health. 2025 Jul 31;2(7):e0000339. doi: 10.1371/journal.pmen.0000339 (PMC12798438; doi:10.1371/journal.pmen.0000339)
Supplement: S1 Fig — (DOCX) [file pmen.0000339.s001.docx]

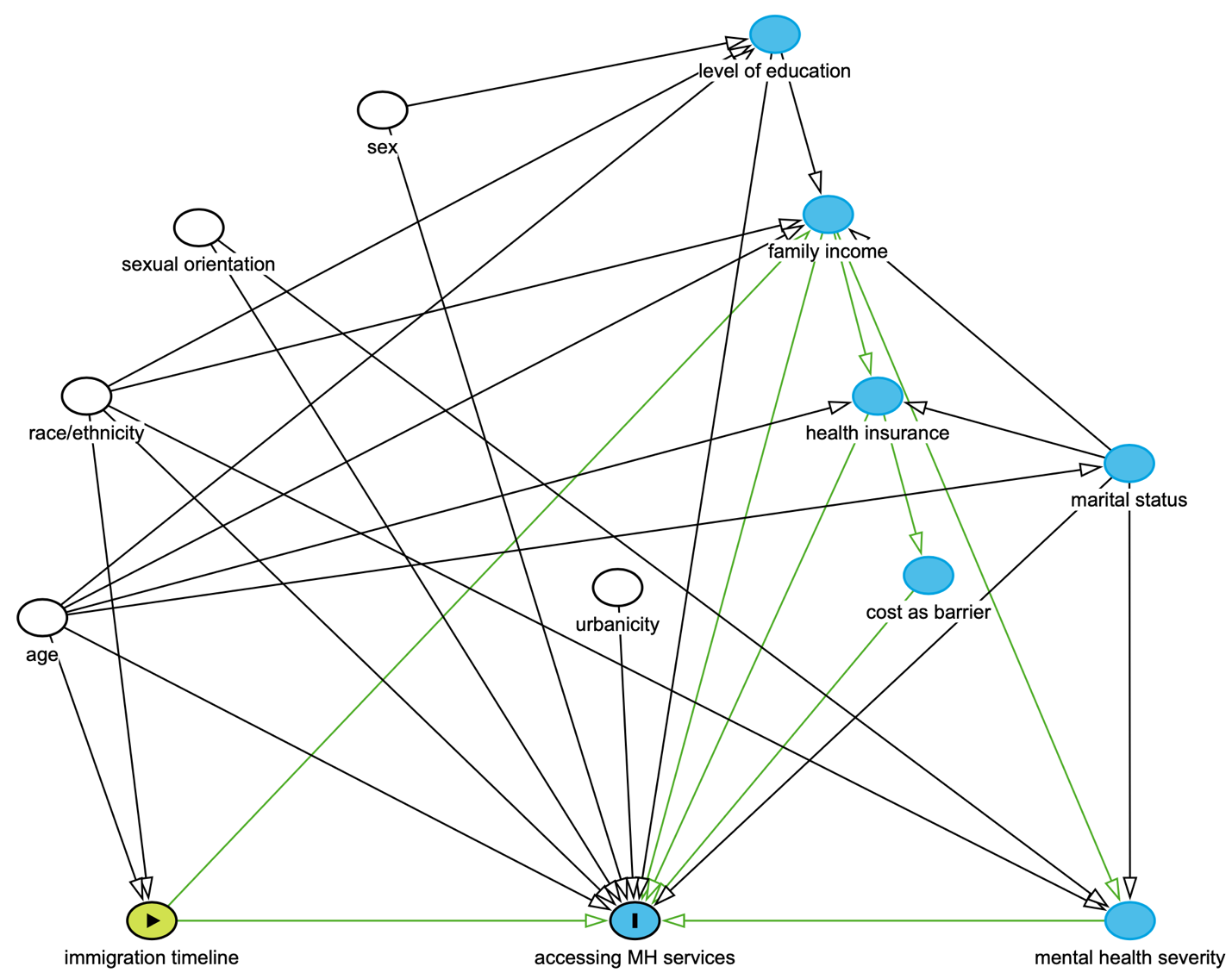


**S1 Fig. Directed acyclic graph (DAG) of the relationships between immigration timeline and mental health service use.** Covariates considered for adjustment include demographic, socio-cultural, financial, and geographic factors influencing ability and willingness to engage in treatment. The green node represents the exposure, and the blue node the outcome. White nodes represent the minimum sufficient adjustment set to account for confounding, plus sexual orientation, sex, and urbanicity as risk factors.
