# Supplementary material for: Accessing mental health care: A population-level exploration of the impact of immigration duration in the United States 2019–2023
Source: PLOS Ment Health. 2025 Jul 31;2(7):e0000339. doi: 10.1371/journal.pmen.0000339 (PMC12798438; doi:10.1371/journal.pmen.0000339)
Supplement: Attachment — Submitted filename: PLOSMH_Response to Reviewers.docx [file pmen.0000339.s003.docx]

Wenjie Duan, Ph.D.

Academic Editor PLOS Mental Health

May 06, 2025

**Re: Accessing mental health care: A population-level exploration of the impact of immigration duration in the United States 2019-2023, Submission ID: PMEN-D-25-00049**

Dear Dr. Duan,

Thank you for your correspondence dated March 4, 2025. We appreciate the thoughtful feedback we received from the reviewers regarding the manuscript that we submitted for possible publication in *PLOS MH*.

As requested, we have revised the manuscript in light of the comments we received. In particular, we have added passages to improve the clarity of our methods and more thoroughly discuss the role of cultural norms in mental health care engagement. In the appended document we have outlined our responses to the reviewer comments and have distinguished the text that was added to the manuscript in bold font. A copy of the revised manuscript is also enclosed with tracked changes.

We found the comments of the reviewers to be very helpful and believe that the manuscript is greatly improved as a result. We have sought to be highly responsive and to clarify our reasoning behind every decision.

We hope you find these revisions to be acceptable. Should there be any other information you require, please do not hesitate to ask.

Thank you again for your consideration,

Suiqiong Fan

PhD Student, MScPH

School of Population and Public Health, University of British Columbia

2206 East Mall, Vancouver, BC, Canada, V6T 1Z3

**Reviewer 1**

1. *Great work, indeed, especially in the light of the new US policy related to migration. The article is well written, with no need for further adjustments. I do hope that it will be published as soon as possible.*

We sincerely appreciate reviewer 1’s encouraging feedback. We are pleased that the reviewer found our work well-written and relevant to current US migration policy.

**Reviewer 2**

1. *Article analyzes the association of number of years of residence in US and mental health service use, i.e. recent immigrant versus established immigrant. Strengths of the study is the nationally representatively sample, although, limitations of language, is one significant limitation which the authors acknowledge. Authors found that one in five respondents (21.2%) reported currently accessing mental health services. Recent immigrants (<5 years) had 46% lower odds of being on medication or in therapy compared to those in US greater than 5 years. The paper is clearly written and scientific rationale given for the design of the study.*

We thank reviewer 2 for their supportive comments and recognition of the study’s strengths, including the use of a nationally representative sample. We acknowledge the reviewer’s point regarding language limitations as a significant constraint and are pleased that the rationale and design of the study were clearly conveyed.

2. *Most American who are not immigrants do not use mental health services even when they have need. In order to have an understanding of what the study results mean, can authors give information about the percent of non-immigrant populations who use mental health services and also information about use of mental health services in Latin American or countries of origin of the immigrants who are represented in this dataset? That way, the reader can put percent service use by immigrants in the U.S. in context. Agree with the author that mental health is important and services should be provided for all, but the issue is complicated by many factors beyond systemic inequity. They should get access to care, but plugging them into traditional mental health services may not be effective, given cultural barriers.*

We thank reviewer 2 for their comments and agree that access to mental health care encompasses a number of factors alongside systemic inequity. To position our findings within the wider context of mental health service use in the United States, we have cited relevant literature comparing mental health services use between immigrants and the US-born population at line #71:

**Estimates suggest that 16% of the general US adult population receive mental health services regardless of need, with lower rates reported among immigrant populations.[4,6] This holds true within specific racial/ethnic groups as well.[4] For example, among African Americans and Caribbean Blacks, those born in the US are almost four times more likely to receive mental services, including formal and informal care, than those born overseas (19% versus 5%).[7]**

Further, to provide a direct comparator using the same methods and dataset, we have additionally provided descriptive statistics detailing mental health service engagement among domestic born Americans with monthly or more feelings of depression and/or anxiety. This led to the following additions to the Methods (line #132; #190), Results (line #238), and Discussion (line #316):

**All analyses were restricted to immigrants born outside of the US, apart from the estimate of mental health service utilization among domestic born Americans.**

**Descriptive statistics estimated the weighted proportion of non-immigrant, domestic born Americans with monthly or more feelings of depression and/or anxiety who used of mental health services.**

Among them, 1,447 participants (21.2%; percentages present weighted proportions) reported currently accessing formal mental health services, **compared to 36.9% among the domestic born population.**

One in five **immigrants** (21.2%) reported currently accessing mental health services, **compared to 36.9% of the domestic born population.**

3. *A factor the authors don’t consider is whether recent immigrants have conceptualizations of mental health symptoms and treatment that are aligned with biomedical concepts in the U.S. that would facilitate service use. The cultural issues are not discussed enough, such as cultural and explanatory models that influence identification and help seeking for any health problem. There are many non-immigrants who do not believe in help seeking for mental health. This is even more the case in immigrant populations. Authors state that recent immigrants do not seek physical health services according to need so that casting the results only in the context of inequity is simplistic. In a new country, not speaking the language and unfamiliar with how American society and healthcare works, recent immigrants will have difficulty using what is available. Before thinking of tailoring interventions, we need to understand how they think about mental health and what they will accept for intervention.*

We appreciate reviewer 2 for re-centering these important considerations. The following was added to the Discussion on line #343 to more explicitly state the role of cultural differences on conceptualizations of mental health and subsequent treatment seeking patterns:

In addition to these economic and structural barriers, the observed disparities might also reflect differences in care-seeking behaviours across immigrant subgroups. **Previous research has highlighted how cultural conceptualizations of mental health and treatment avenues influence immigrants’ engagement with formal services. These include tendencies towards informal mental health support systems such as family, friends, and religious or spiritual advisors.[4,54] As the present analysis suggests, many of these influencing factors may diminish over time as immigrants acculture to the US.[54] However, socio-cultural barriers to mental health services use such as experiences of discrimination, stigma, and lack of trust in the healthcare system further define US immigrants’ engagement with formal services.[8,33,53]**

**Reviewer 3**

1. *This manuscript is well written, interesting, topical and relevant for readers of PLOS Mental Health. It does require a major revision and additional work to address limitations, and additional detail in certain areas.*

We appreciate Reviewer 3’s extensive effort and comprehensive review. We have revised our manuscript to address their comments.

2. *Key terms are not defined fully at first usage. Examples include "over time" (calendar time? time since immigration?), "immigration", "immigrants" (seems obvious but a formal definition is needed), "medication or counselling", "care", "culturally safe", "stigma". The meaning is often clear but not necessarily - all key terms must be defined specifically, at first usage. Remember that readers outside US/Canada won't know what typically happens when people with psychological distress seek help - who pays, what is available, what is typically offered?*

We thank reviewer 3 for highlighting the areas that require clear definitions. We have edited the manuscript to improve clarity of key terms and provide more insights into the US’ mental health care system. Commonly used terms such as “immigrants” and “medication or counselling” are defined in the methods section to specify how the concepts are being measured as variables in our analyses.

3. *There is confounding with period effects i.e. trends in mental health across 2019 to 2023 in the wider population (not just immigrants) and also from the COVID-19 pandemic which impacted mental health. Domestic born adults are excluded entirely, so it is difficult to determine what is a period effect, and what is an immigration effect. Table 1 suggests there is increasing common mental distress increasing over time. Can you address this in a revision, by comparing to the wider population and proposing a way to separate the two effects?*

We thank the reviewer for raising this important point. As outlined in the Introduction, most prior studies on immigration and mental health focus on comparisons between immigrants and U.S.-born individuals. As a result, in our study, we specifically aimed to explore how time since immigration influenced mental health service utilization among U.S. immigrants, to fill an identified gap in the literature. Revising the present analysis to include comparisons to domestic born Americans is thus beyond the objective of this manuscript. However, we have provided descriptive statistics of service engagement among non-immigrants to provide a direct comparator given the diversity in methods behind these estimates in the literature (please see our response to reviewer 2’s comment #2).

Regarding the possibility of confounding by period effect, the inclusion criteria of monthly or more feelings of anxiety and/or depression precludes the possibility that an underlying trend in mental health rates would confound our results on mental health service use. Nonetheless, we agree that the COVID-19 pandemic may have impacted mental health service use. To examine this, we conducted a sensitivity analysis stratified by pandemic period (pre-COVID, COVID, and post-COVID), as presented in Figure 2 and described in the Results section. This analysis showed that the association between recent immigration and reduced service use persisted across time periods, supporting the robustness of our main findings.

Finally, we wish to clarify a potential misinterpretation. Table 1 presents the proportion of immigrants using services, not prevalence of mental distress. The increase over time reflects non-significant variation in service use rather than an increase in reported distress levels, which are not a focus of this analysis.

4. *The five year effect is positioned as the period of interest for the main results, but is confounded with Medicaid eligibility. Shouldn't the follow-up period start after five years? If care cannot be accessed during the first five years, it will distort the data in this period. The research question concerns immigration timeline in relation to mental health service use, so this is major flaw in my view in the current version. I accept the first five years are a 'critical period', but if access to services is restricted until after five years, this should not be used as a cut-off. I would have started follow-up after five years (5 to 15+ years), assuming that services are less available before that time. First five years could/should be a sensitivity analysis, in my view.*

We appreciate the reviewer for raising this as a concern. In the United States, a majority of people have private health insurance through their employers. Among the general population, 2021 estimates found that 36% of people are covered under public insurance plans, while 66% hold private insurance (categories are not mutually exclusive) and 8% are uninsured (Keisler-Starkey and Bunch (2022) Health Insurance Coverage in the United States: 2021). This means that care can be accessed by immigrants, with coverage, before the 5-year mark. To provide greater clarity on this point, we have added the following text to line #152:

The five-year cut-off was chosen for three reasons. Firstly, the first five years are widely considered a critical period for new immigrants as they adjust to a new cultural, social, and economic environment. Moreover, **while immigrants can have private insurance provided by employers, they can additionally qualify** for certain social services, including some forms of healthcare assistance, after five years.

Within this context, we view the eligibility criteria for health insurance such as Medicaid to relate back to the systemic inequities faced by immigrants that we explore in the Discussion.

5. *It is not clear why those without symptoms of psychological distress were excluded entirely. By excluding domestic born and those without distress, important comparator groups are missing.*

As discussed in the introduction, the prevalence of mental health concerns is known to increase the longer immigrants are in their adoptive countries. If the sample had not been restricted to immigrants who reported at least monthly feelings of depression and/or anxiety, this underlying trend would have obscured the association we aimed to assess. The following text was added to line #126 to elucidate this point:

Adult participants (≥18 years) were eligible for the present study if they were born outside the US and reported daily, weekly, or monthly feelings of depression and/or anxiety (defined as feeling worried, nervous or anxious). This **criterion was included to ensure the sample was eligible to experience the outcome, and was** based on the Washington Group anxiety/depression indicator category, which classifies less than monthly symptoms as no anxiety/depression.[22]

Please refer to our response to comment #3 regarding the exclusion of domestic born Americans.

6. *Missing data has been considered, but could be simplified. Typically missing data on exposure/predictor or outcome is not allowed, but missing data on covariates can be imputed. Multiple imputation with chained equations rarely produces more biased estimates than deleting cases with missing covariate data. In my view it would have been sufficient to define the analytic sample as nonmissing exposure, nonmissing outcome - then impute on covariates. Rather than presenting complete case analysis as the primary (P8L196). Imputation on exposures is more controversial.*

We appreciate that there are numerous approaches to handling missing data and thank reviewer 3 for their thoughts on the matter. Indeed, imputing the exposures is a more controversial, but nevertheless acceptable, approach to multiple imputation when using MID. Although we only had relatively small numbers of missingness in the outcome (n=12), we followed this most acknowledged approach for our imputation to avoid unnecessary sample exclusions. We have added justification to our Methods (line #208) with reference:

**Multiple imputation then deletion analysis is recommended for data with missing information in the exposure, outcome, and covariates, especially when the exposure/outcome can inform the covariates to impute.[44]**

7. *Less common and more serious mental disorder is not included (psychosis, suicidal ideation etc.) which needs emphasising more in the discussion section.*

In accordance with the reviewer's request, the following was added to line #399 to reemphasize the fact that this analysis is focused on anxiety and depression:

Contrastingly, a US-based study using 2001 to 2005 data found no association between years in the US and mental health treatment.[60] These diverging findings may in part be due to [...] **Finally**, they utilise a stricter inclusion criterion (severe mental health diagnosis) and a longer outcome window (treatment in the last 12 months), which might smooth out the utilization differences between recent and established immigrants. **These diverging results highlight potential areas of future research given the present analysis did not capture less common, but potentially severe, mental health disorders.**

8. *The impact of "intersectional" identifies needs more attention as a limitation and area for future research. For example, in Table 1 there are nearly twice the prevalence of symptoms in lesbian/gay/bisexual adults compared to heterosexual. Do immigrants with minority ethnic and minority sexual identity experience hostility or prejudice (e.g. racism from White/domestic LGBs), contributing to worsening symptoms over time? Perhaps find some work from "All of US" or "Pride study" for any insights here, in the discussion section. As currently written, readers might assume that the structural factors are creating psychological distress, but it could be hostility from other minority groups as well.*

We thank the reviewer for the comments on intersectionality. We have added a paragraph in the Discussion to expand on the role of intersectionality and within-group heterogeneity (line #354):

As the present analysis provides a high-level view of mental health service engagement among US immigrants, it is important to **acknowledge the considerable heterogeneity within immigrant populations.[4,5,32] Future analyses are needed to better understand how specific subpopulations of immigrants seek, and experiences barriers to, mental health care. For example, a 2023 ecological study found that having a higher concentration of Latin American or Asian immigrants was linked to fewer mental health visits overall, while the concentration of European immigrants showed no association[55]. These analyses would further benefit from using intersectional approaches, whereby overlapping and intersecting identities are considered when investigating trends in service engagement and barriers to care. [56,57] Immigrants are not a monolithic group, and factors such as country of origin, legal status, language proficiency, socioeconomic background, gender, and sexual orientation interact to influence mental health service use.[4,5,32]**

With regards to their specific example, we suspect the reviewer may have misinterpreted Table 1, as the prevalence of mental health symptoms by sexual identity is not presented. Rather, Table 1 shows that immigrants who identify as a sexual minority had higher rates of service engagement compared to straight respondents (113/267 vs 1,307/5,816). However, we do not go into depth with our interpretation of this association as sexual identity was conceptualized as a confounder in our study.

9. *The status of study variables is not currently clear as currently written. On P6L151 onwards, variables are positioned as confounders/covariates. But mental health severity is later positioned as an effect modifier. It is confusing to read, so I recommend a separate paragraph describing effect modifiers. Best practice is to pre-register all of this in advance on Open Science Framework (OSF), otherwise it can read as rather exploratory.*

We appreciate that we did not adequately explain the status of variables considered in this analysis. To improve clarity, the Methods section was amended to distinguish between variables that were considered in the DAG, and variables that were ultimately controlled for in the model.

10. *Relating to this, the registration of OSF does not appear to have research questions or analysis plans, and was done in January 2025. It looks like the OSF registration is to faciliate data sharing, which is good, but really the whole project should have been pre-registered for full transparency.*

In effect, the OSF registration was a measure taken to improve the transparency and reproducibility of our analysis, and did not include a pre-registration of the analysis plan. To acknowledge this, the following was added to line #415 in the limitations section:

However, there are several limitations to consider. First, **as the analysis plan was not registered prior to its undertaking, findings should be considered exploratory and independently substantiated in future studies.**

11. *Related to this, it is perplexing to have symptom severity as an effect modifier and part of the outcome/dependent variable (more severe symptoms are more likely to become eligible for counselling/medication), particularly when subgroups/modifiers were not pre-registered and delineated. Aditionally, there are structural zeros in Table 1 (100% because not using services means they are no medicated, in counselling etc.). To simplify all this analysis, can an outcome variable be created that captures service use intensity and severity of symptoms somehow? Consider creating groups (multinomial logistic regression) or assigning 1 point for more "intense" distress (e.g. most distressed is anxious, depressed, medicated, in counselling). Consider bringing back in the no distressed group (0 points) and domestic-born (to capture period effects in distress in the wider population, see point above). I found the current approach a little difficult to follow, and had to re-read parts of the manuscript in order to understand what was happening, which is not a good sign. If I had to, then so will readers...*

We thank the reviewer for their thoughtful comments. Symptom severity was considered as an effect modifier for the reason described by the reviewer (i.e., people with more severe depression/anxiety are more likely to need, seek, and engage with mental health services). Since our main analysis’ inclusion criteria is very broad (i.e., monthly or more feelings of depression/anxiety), we sought to explore whether the relationship between immigration timeline and service utilization differed based on symptom severity. This was considered important for policy implications, as well as to explore whether recent immigrants have a different threshold of severity required to access services than more established immigrants. While we appreciate the reviewer’s creative suggestions for a more layered analysis, service use intensity (in terms of frequency of access, length of access, etc.) is not captured in NHIS data.

Please refer to our responses to comments 3 and 5 for an explanation around why we did not include domestic born Americans and people with no depression/anxiety, respectively, in the analysis.

12. *I'm not convinced the DAG (Figure 1) is necessary, given that the IV, DV and covariates are clear (and effect modifiers are specified separately). It's a fairly standard regression not a path or structural equation model.*

We appreciate the reviewer’s suggestion for simplifying the presentation of our manuscript and agree it is not required in the main body of the paper. However, as we believe the DAG improves the legibility of how we identified the minimum sufficient adjustment set and risk factors for the final model, we've elected to move Figure 1 to the supplementary materials.

13. *The study population excludes those who wouldn't be captured into the survey. Many immigrants will be undocumented and even if approached by the interviewer, might think the interviewer represents the government which would increase the probability of nonresponse/refusal. Even if someone in the household was interviewed, there may be others in the household who are not captured in the data for these and other reasons. This needs additional attention in the discussion section as a limitation.*

We thank the reviewer for raising this important limitation. In accordance with the proposed revision, the following was added to the Limitation section (line #450):

**Similarly, though immigrants with precarious status, including undocumented immigrants, were not precluded from participating in the NHIS, they likely had high rates of participation refusal that may not be well accounted for by the survey weights, further limiting generalizability to all immigrants.**

14. *The healthy migrant effect is referenced a few times in the manuscript, but I am skeptical about this. Data linkage research has shown that data linkage and data quality issues bias not just the effect sizes but also the directionality of estimates when comparing White with Hispanic groups. Healthier, wealthier Hispanic populations are more likely to be linked in adminstrative data (and presumably in survey design) so they are be compared with White (not all Hispanic vs White).* [*https://pmc.ncbi.nlm.nih.gov/articles/PMC4598042/*](https://pmc.ncbi.nlm.nih.gov/articles/PMC4598042/)

We thank the reviewer for highlighting this as a possible concern. In the article cited, it appears that inaccuracies in the comparison of White and Hispanic mortality rates comes from data quality issues in the process of record linkages. As our analysis relies solely on NHIS data, this should not be a concern. As a nationally-representative survey of the non-institutionalized American population, the NHIS has integrated processes to account for potential biasing effects like non-response rates that are then accounted for in our models via weight adjustments.

15. *Please consider that among Hispanic populations, the culture is more allocentric and this might impact norms around how mental health symptoms should be addressed. Psychotherapy and medication is more common in more individual centric cultures, but for many Hispanic adults the wider family could be said to be "the patient" and more senior family members might traditionally have been involved/consulted before seeking psychotherapy/medication. Might ties with extended family be part of the mechanism here, if there are fewer family support resources to draw on in the new country?*

*We acknowledge that cultural differences play an important role in how people understand and navigate mental health challenges, including accessing formal care. Please refer to revisions made in response to comments #3 from review 2 regarding the role of cultural norms around mental health and informal support systems. Exploring informal mental health supports was beyond the scope of our research question, which is focused on formal mental health services, in part due to the limitations of the NHIS dataset.*

16. *Finally, was there any data on alcohol, tobacco and drug use? These are relevant covariates and perhaps partial mediators.*

We thank the reviewer for raising the possibility of substance use variables being important to our model. We agree that substance use may be associated with different patterns of mental health service utilization, through diverse mechanisms including shame, stigma, and the management of comorbidities. However, substance use cannot theoretically be understood to proceed, or have an effect on, immigration timeline (i.e., how long since someone immigrated to the US). Rather, if there is an association between the two, the association is likely driven by length of stay in the United States on substance use patterns. Within our analysis, this would make substance use a mediator and should thus not be included in the model.

***MINOR ISSUES TO BE ADDRESSED***

*M1. Abstract: although I appreciate the survey design was a complex survey design, it is not necessary to mention this in the abstract which is distracting. I suggest describing it as a representative population survey of [population]. If the survey design features are addressed in the model, which they are, it is representative. Readers unfamiliar with complex survey designs will scan the abstract and interpret 'complex' to mean the study is 'complicated', a different meaning. Representing 11.9 *adults* annually.*

As suggested by the reviewer, we have amended the language in the Abstract (line #36):

Using 2019–2023 National Health Interview Survey data, this **population-based** study examines how time since immigration influences use of mental health services among immigrants reporting monthly or more depression or anxiety symptoms **among civilian, non-institutionalized adults in the United States**. Of the 6,201 participants (representing 11.9 million **adults** annually)...

*M2. P3L67 Are you saying that the apparent advantage (which may not be real - see my point above about bias) not only lessens over time, but becomes worse that domestic born populations? That is not clear, so needs rewriting for clarity.*

To address the reviewer’s comments regarding the clarity of the text in the Introduction, we have rewritten the following sentence (line #65):

This decrease in immigrants’ health over time is well in line with the wider literature on the Healthy Immigrant Effect (i.e., whereby recent immigrants have better health than the domestic born population upon arrival, **though the advantage is lost over time, eventually matching the domestic-born population or lower).**[3]

*M3. P4L83 You need to compare this figure to the increase observed in domestic born populations and White groups. Again, a possible period effect.*

We agree with review 3 that the cited increase in psychological distress among immigrants between 2015 and 2021 is likely reflecting a period effect. Unfortunately, the policy brief cited does not provide comparative statistics for the domestic born population of California. However, we still believe the reference provides value by depicting the potentially growing importance of understanding mental health trends and subsequent unmet healthcare needs among immigrant populations in the United States. Further, we would posit that using “White groups” as a unique comparator is not appropriate for this point as immigrant and domestic born populations both encompass a wide variety of racial/ethnic groups.

*M4. P6L153 Was "straight" the exact wording in the interview? Was it self-completion or an interviewer asking this? Would potentially impact response.*

We made concerted efforts to keep the language used in the manuscript consistent with the NHIS, including their use of the category ‘straight’ in their sexual identity variable. In response to the reviewer’s concern with the effects of NHIS being an interviewer-administered survey, we have added the following to our Limitation section (line #419):

**Third, the interviewer-administered and self-reported nature of the data introduces the potential for social desirability and recall bias.**

*M5. P16L317 See point earlier about "Healthy Immigrant Effect". This may be biased if healthier subgroups having better data quality are more likely to appear in health records that get linked together correctly, at an earlier time. Missed matches in administrative data are much more common for minority groups - they are more likely to get new IDs each time, so records containing detail are more likely to be missing.*

Linked administrative data were not used in the present study, and there were no repeat observations from the same individuals. All data originated from the NHIS, an annual, cross-sectional, interviewer-administered survey.

*M6. P16P327 You mention undocumented migrants here, but more needs to be made of this limitation. You have probably underestimated the main effects because healthier, wealthier immigrants make it into the interview/survey. It might be as repsentative as possible, but will miss those in the household reluctant to take part, fearful, or those 'off the radar'.*

We agree with the reviewer’s concerns and amended the Limitation section to acknowledge this point. Please refer to our response and revisions described above, in comment 13.

*M7. P17L350 Reason for immigration is an important limitation, needing more acknowledgement. For example, war, human traffic, slavery - quite different from employer sponsored work permit etc. Lots of heterogeneity.*

We agree with the reviewer that this is a limitation of our analysis, as is outlined in our limitations section. To highlight the need for more research on this topic, we added the following to the Limitations section on line #427:

Forth, there may be unmeasured confounding in the analysis. Potential confounders that were not collected as part of NHIS and thus could not be assessed include country of origin (where race/ethnicity could be considered a proxy) and type of immigration (e.g., work permit, family sponsored, refugee, undocumented). However, the direction and the magnitude of this potential bias is not predictable. Though only minimally related, immigration pathway was considered via legal status by investigating whether the effect of immigration timeline was modified by citizenship status. No such effect modification was found. **Future analyses should explore how type of immigration influences service utilization and barriers to care.**

*M8. P18L367 My point about period effects in the study period is relevant here, but downplayed too much. In the current version of the manuscript it is a major flaw. This needs to be addressed. The socio-structural changes impact the entire population. Aditionally, there was hostility in media/politics to immigration accelerating since 2005 which might confound with the "time since immigration" pattern in the data.*

Please refer to the previous response (#3) where we highlight our sensitivity analysis investigating a potential period effect on the outcome surrounding the COVID-19 pandemic, and justify the exclusion of a domestic-born comparator group in the analysis.

*M9. P18L376 "imputation for missing data" - on what? Be clear here - exposure/covariates?*

We have amended the relevant sentence to re-specify the MID approach used (line #412):

Sensitivity analyses, including **a** multiple imputation**, then deletion analysis** for missing data and an alternative categorized exposure variable, ensure the findings’ robustness.

*M10. P20L413 Do you mean "not statistically significant", "was insignificant" does read correctly.*

We agreed with the reviewer and have changed the language accordingly (line #459).

*M11. Table 1. Are all these figures weighted for survey design weights? If not, they should be.*

As highlighted as necessary by the reviewer, all analyses account for survey weights, as stated in the footnotes of our tables and figures.

*M12. Among those with health insurance covered, 91.4% are using services, which is very high. What is happening here? For readers outside US/Canada, we need to understand how mental health services are usually made available, and the role of voluntary/community/third sector organisations. If services are inaccessible without medicare/insurance, then the five year window is problematic as mentioned above.*

Among people accessing mental health services, the 91.4% in table 1 refers to the percentage of people who reported having health insurance compared to 8.6% who are doing so without coverage. Service use among people with health insurance coverage is much lower, at 26% (in the sample [1,345/5,232], not accounting for survey weights).

Please refer to our response to comment #4 regarding the role of private versus public health insurance in the United States, and response #3 to review 2 discussing informal mental health supports. Given limitations in the NHIS dataset, we were not able to explore the role of informal or alternative mental health supports (“voluntary/community/third sector organisations”), as we further discussed in our Limitations (line #435):

Further, the variable is not inclusive of alternative mental health supports such as religious or community-based organizations. These may serve as low-barrier alternatives to medications and therapy; providing accessible and culturally relevant mental health support.[4] **Informal mental health support can be particularly important in collectivistic cultures, where immigrants tend to first seek support from friends, family, and the community.[64,65]** These informal services could explain some of the observed disparities though data were not available in the NHIS to explore this hypothesis.

*M13. The 100% values for medication and counselling/therapy are structural zeros (see above). Should they be N/A?*

As suggested by the reviewer, the structural zeros in table 1 have been replaced by “N/A.”
